# Bilateral congenital lobar overinflation (CLO) – a rare presentation of an uncommon condition^☆^

**DOI:** 10.1016/j.radcr.2022.07.020

**Published:** 2022-07-27

**Authors:** Abenezer Zinaye, Abrehet Zeray

**Affiliations:** Department of Clinical Radiology, College of Health Sciences, Faculty of Medicine, Addis Ababa University, Addis Ababa, Ethiopia

**Keywords:** Congenital lobar overinflation, CLO, Congenital lobar emphysema, Bilateral CLO, Neonatal respiratory distress

## Abstract

Congenital lobar overinflation (CLO) is a rare congenital pulmonary abnormality characterized by progressive overexpansion of a lobe(s) exerting mass effect on the remaining lobes and the mediastinum. Bilateral involvement is extremely rare and has been reported only a handful of times. We present a case of bilateral congenital lobar overinflation in a term female neonate managed with single-step bilateral lobectomies.

## Introduction

CLO is a developmental pulmonary abnormality characterized by progressive overinflation of one or more lobes of the lungs with resultant mass effect and compression of the remaining lobes [1]. The cause is not clearly elucidated as of yet, but it is thought to be due to either intrinsic cartilaginous abnormality (absence or weakness) or extrinsic narrowing of the airway by a mediastinal mass such as a bronchogenic cyst or a vessel. There is an established lobar predominance with the most commonly involved lobe being the left upper lobe, followed by the right middle lobe and right upper lobe. Lower lobe involvement is rare [2]. Bilateral involvement is even rarer with fewer than 20 reported cases throughout modern literature. A summary of the reported cases of bilateral congenital lobar overinflation is listed in Table 1. The vast majority of the reported cases showed involvement of the left upper lobe and right middle lobe.

**Table 1:** Previously reported cases of bilateral congenital lobar overinflation.

Authors	Year of report	Involved lobes
Floyd FW, et al. [6]	1963	LUL, RML
May RL, et al. [7]	1964	LUL, RML
Schramel R, et al. [8]	1968	LUL, RML
Schiller HM, et al. [9]	1970	RML, RLL, LLL
Tournier G, et al. [10]	1973	LUL, RML
Ekkelkamp S, et al. [11]	1987	LUL, RML
Stigers KB, et al. [12]	1992	2 cases (RML, LLL and RLL, LLL)
Maiya S, et al. [13]	2005	LUL, RML
Kumar TS, et al. [14]	2006	LUL, RML
Abushahin AM, et al. [15]	2012	LUL, RML
Perea L, et al. [4]	2017	4 cases (all LUL, RML)
Ait Chtouk M, et al. [16]	2020	LUL, RML
Lei Q, et al. [17]	2020	LLL, RML
Sawant V, et al. [5]	2021	LUL, RML

We report a rare case of bilateral CLO involving the left upper lobe and right middle lobe in a female newborn presenting with respiratory distress since birth. She was successfully managed with single-stage bilateral lobectomies. As to our search, this is the first reported case of bilateral CLO in Ethiopia.

## Case report

**Fig. 1:**
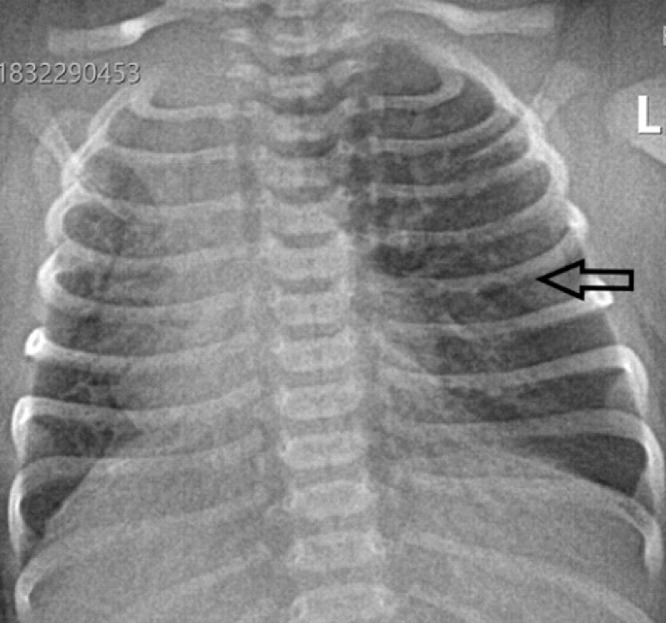
Frontal chest radiograph showing hyperinflation of the left lung (arrow) with mediastinal shift to the right side.

We report a case of a 28 days old female neonate who was born at a gestational age of 39 weeks and 4 days by caesarian section for an indication of 2 previous CS scars. She presented with fast breathing since 6 hours of age which progressively worsened over time. At presentation, she was tachypneic (65 breaths per minute). She also had subcostal/intercostal retractions and mild chest in drawing. Chest auscultation at initial presentation was unremarkable. Baseline investigations including CBC, RBS, RFT and serum electrolytes were normal except for a mild hypokalemia which was corrected. Blood culture was non-revealing. Abdominal ultrasound was also normal. Transthoracic echocardiography (not shown) revealed patent foramen ovale with left to right shunt. She was admitted with a working diagnosis of EONS. She failed to improve despite being put on IV antibiotics and intranasal oxygen. Chest radiograph and subsequently chest CT were obtained. The initial chest radiograph (Fig. 1) revealed hyperinflated left lung with mediastinal shift to the right side. Two possibilities were initially considered, the first being right lung hypoplasia with compensatory hyperinflation of the left lung. The second differential consideration was left lung hyperinflation with a vascular ring as a possible cause. Chest CT was then obtained to better characterize the abnormality detected on the chest radiograph.

**Fig. 2:**
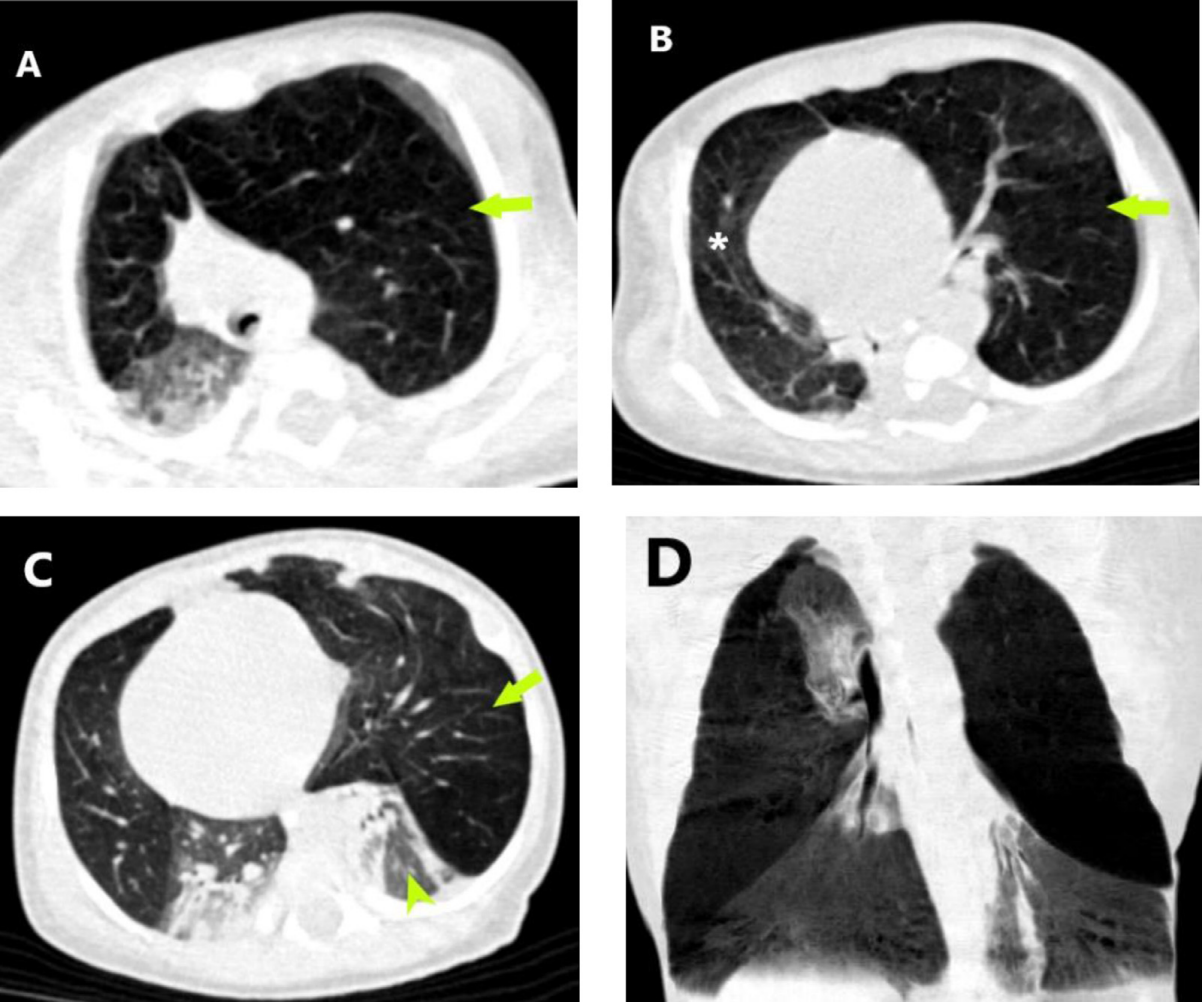
Axial chest CT images in lung window settings at different levels (A through C) demonstrate the presence of marked overinflation of the left upper lobe (arrows) resulting in compressive atelectasis of the left lower lobe (arrowhead) and significant mediastinal shift to the right. Right middle lobe hyperinflation (asterisk) with collapse consolidation of the right upper and lower lobes is also seen (C). Coronal minimal intensity projection (D) better demonstrates the overinflated and collapsed lobes.

The chest CT obtained at the age of 28 days (Fig. 2) revealed the presence of marked hyperinflation of the left upper lobe with attenuated pulmonary vasculature and causing significant mediastinal shift to the right side. In addition, the right middle lobe was also hyperinflated demonstrating similar attenuation to that of the left upper lobe. Compressive atelectasis of the remaining lobes (bilateral lower lobes and the right upper lobe) was noted. On the mediastinal window, no compressive mass or enlarged vessel was demonstrated.

**Fig. 3:**
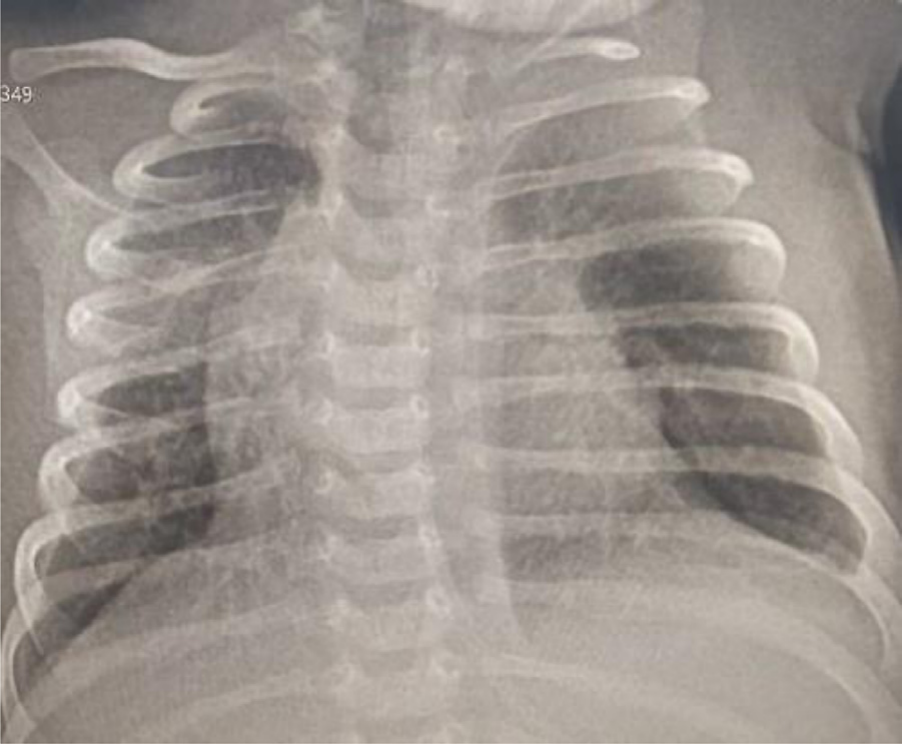
Postoperative frontal chest radiograph shows expansion of the previously collapsed lower lobes and the right upper lobe, and resolution of the mediastinal shift.

The diagnosis of impending respiratory failure secondary to bilateral congenital lobar overinflation was made, and the infant was prepared for surgery. Thoracotomy and single-stage bilateral lobectomy of the left upper lobe and right middle lobe was performed. She had a smooth postoperative course. A chest radiograph obtained on her seventh postoperative day (Fig. 3) showed optimal expansion of the previously collapsed lobes and resolution of the mediastinal shift. The infant was discharged on her tenth POD. She is being followed at outpatient clinic and is doing well as of the time of writing this case report.

## Discussion

Congenital lobar overinflation, also known as congenital lobar emphysema is an uncommon abnormality of the lungs characterized by overinflation of a lobe. It is thought to be due to an intrinsic or extrinsic bronchial abnormality causing a ball-valve mechanism with resultant hyperinflation of the affected lobe [3]. CLO classically presents in the neonatal period with respiratory distress, with 80% of cases presenting by the age of 6 months. It is 3 times more common in males (M:F = 3:1) [2]. The left upper lobe is the most frequently involved lobe (42%) followed by the right middle lobe (35%). Lower lobe involvement is quite rare. Bilateral CLO is also extremely rare with fewer than 20 case reports found in modern literature. As to our knowledge, the case we are presenting is the first of its kind reported in Ethiopia.

Imaging plays an important role in the diagnosis of CLO. Radiographs obtained soon after birth may show opacity of the involved lobe due to retained fetal fluid. Subsequent films will demonstrate hyperlucency and hyper expansion of the affected lobe with associated mediastinal shift and compression of the remaining lobes. CT will better demonstrate the extent of involvement and degree of mass effect. CLO appears as a hyperexpanded lobe with attenuated pulmonary vasculature. CT will also exclude secondary causes of lobar overinflation such as compressing mediastinal masses and vascular anomlies [3]. The vast majority (88%) of the few case reports of bilateral CLO showed involvement of the left upper lobe and right middle lobe. Our case is also in keeping with this pattern with these 2 lobes involved. Given the rarity bilateral CLO, it can be a diagnostic challenge and imaging, especially CT, plays an irreplaceable role in ascertaining the diagnosis. Our patient was not diagnosed until 28 days of age despite having been symptomatic since day 1.

The management of bilateral CLO has been a topic of debate. There are 2 major schools of thought. The first one involves performing a staged surgery with removal of one of the affected lobes (typically the one with the more pronounced mass effect). The child would then be followed and a second surgery to remove the other lobe would be done if indicated. If the child remains stable and there is no significant rebound hyperinflation of the non-removed diseased lung, conservative management would be sought. The rationale behind this approach is that a single stage bilateral lobectomy is a high-risk procedure with increased risk of complications and postoperative pain. In the reviewed case reports and case series, this approach appears to be the more popular one. In the largest single institution case series on bilateral CLO, 3 of the 4 patients were managed with unilateral lobectomy. They were followed and remained stable without needing a second surgery [4]. The second approach would be performing a single stage bilateral lobectomy. It has been advocated by some institutions [5]. Our patient was successfully managed with a single stage bilateral lobectomy of the left upper lobe and right middle lobe. She had a smooth postoperative course and is doing well at the moment of writing this case report.

In conclusion, bilateral congenital lobar overinflation is an extremely rare cause of respiratory distress in the newborn. Its diagnosis requires high index of suspicion and relies on imaging, typically with chest radiographs and CT. There are 2 main approaches to its management including single step bilateral lobectomy and staged (2-step) surgery.
